# Telehealth for Upper Extremity Conditions: Perceptions of the Patient and Provider

**DOI:** 10.5435/JAAOSGlobal-D-20-00127

**Published:** 2020-09-10

**Authors:** Brian M. Katt, Casey Imbergamo, Daniel Fletcher, Daren Aita, Michael Nakashian, Moody Kwok, Pedro K. Beredjiklian

**Affiliations:** From the Division of Hand Surgery, Rothman Orthopaedic Institute (Dr. Katt, Dr. Fletcher, Dr. Aita, Dr. Nakashian, Dr. Kwok, Dr. Beredjiklian), Philadelphia, PA, and the Rutgers Robert Wood Johnson Medical School (Mr. Imbregamo), New Brunswick, NJ.

## Abstract

The recent coronavirus pandemic has prompted providers to adopt telehealth as a way to maintain contact with their patients on an unprecedented scale. The purpose of this study was to evaluate the perception of care for both patients and physicians using telehealth visits for the management of upper extremity orthopaedic conditions. This study consisted of the analysis of surveys sent to both physicians and patients immediately after the completion of a telehealth visit for an upper extremity condition. Eighty percent of patients responded as “very satisfied” with their encounter. Satisfaction scores were similar for patients seen for a new issue or an existing issue. The use of a video platform was preferable to patients compared with a telephone call. Physicians would have requested a radiograph or offered a steroid injection during a new patient evaluation in 77% of cases. Physicians were less satisfied with the use of telemedicine, particularly when evaluating a new patient. A large majority of patients and physicians alike felt telehealth visits have a role in patient management, acknowledging they would both choose to incorporate “some” of their visits as telehealth evaluations for any particular issue.

Telehealth is an umbrella term encompassing the use of telecommunication and information technology for clinical and nonclinical healthcare services including health administration, provider training, continuing medical education, and access to medical literature. Telemedicine is a subset within telehealth more specifically referring to the use of such technology to provide clinical healthcare services from a distance in an effort to diagnose and treat patients. Telemedicine can be used in all facets of patient care including new patient evaluations, follow-up and postoperative examinations, and remote consultations. The use of telemedicine provides access to patient care while minimizing the burden of travel and time spent away from work or school, thus potentially offering a better cost-effective means of healthcare delivery.

Initially regarded as a modality to reach remote populations with limited access to medical services, its role has recently and rapidly expanded.^1,2,3^ The widespread use of telemedicine has allowed for acceptable levels of uninterrupted medical care during periods of imposed isolation as is currently the situation resulting from the coronavirus (COVID-19) pandemic. During such a crisis, when the risks of close interpersonal contact may outweigh the benefits of a face-to-face visit, telemedicine offers a reasonable alternative. Telemedicine has been successfully used in the management of stroke patients and urologic and pediatric surgical subspecialties, among others.^4,5,6^ Although the advantages of telehealth have been long recognized, widespread use in the field of orthopaedics has not been seen until recently.

The outbreak of the COVID-19 pandemic has necessitated social distancing measures, substantial travel restrictions, and shelter-in-place orders. As such, it has limited the ability of patients to seek care for urgent and nonurgent conditions. To provide accessible and safe medical care, one of the limited options for physicians in this setting has been telehealth. For example, Parisien et al found 63% of orthopaedic departments with residency programs are currently offering telehealth services as an option for orthopaedic appointments. An additional 23% of institutions are currently in the process of establishing telehealth capabilities. Of those, 88% cited the COVID-19 pandemic as the reason for implementation.^3^

The goal of this study was to evaluate the broad use of telemedicine in the care of patients with an upper extremity condition. Our hypothesis was that the perception of care provided using telehealth appointments is highly acceptable to patients and physicians for postoperative follow-up or new patient visits.

## Methods

Institutional Review Board approval for this study was obtained before enrollment. The study was designed as a prospective cohort study completed at a single institution. All enrolled patients completed a telehealth visit for an upper extremity report between March 23 and May 15, 2020. The inclusion criteria for the subjects were as follows: aged 18 years or older, intact decision-making capacity, completion of a new follow-up, or postoperative visit for a report of the upper extremity, access to the technology required to participate in a telehealth visit, and completion of an online survey.

The telehealth encounter was performed by one of the seven fellowship-trained and board-certified orthopaedic surgeons specializing in upper extremity surgery. The mode of communication included either a telephone call or video conference encounter at the discretion of the physician and the technology available to the patient, as well as the patient's ability to use the available technology. Physician options to complete the video conference encounter included common videoconferencing applications such as Apple FaceTime, Duo, or Skype. The Health Insurance Portability and Accountability Act compliant platforms included Doxy.me, the Doximity Dialer, or eClinicalWorks/healow applications.

**Figure 1:**
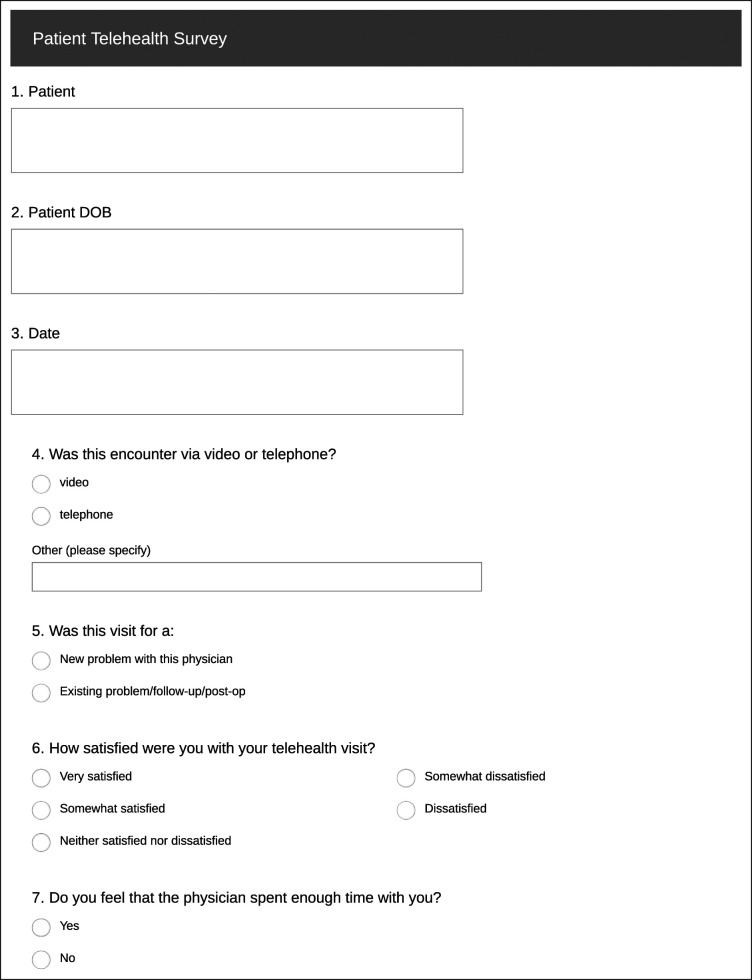

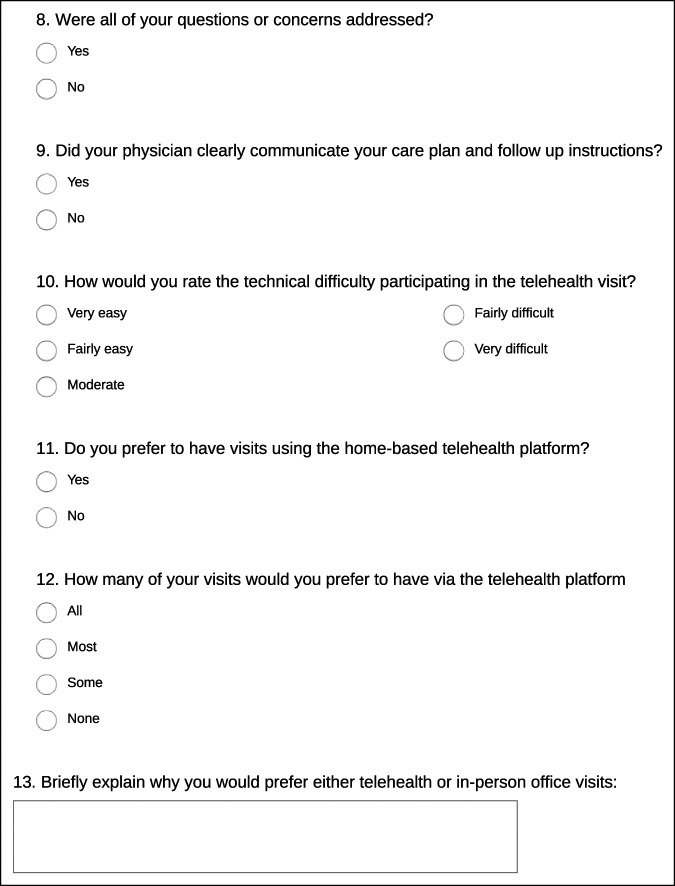
Chart showing patient survey.

This study consisted of two parts. First, a postvisit survey was offered to each patient who completed a telehealth encounter with a participating provider. The patient survey (Figure 1) included questions related to the mode of the encounter, quality of time spent with the provider, preference of telehealth versus an in-office encounter, or a combination of both. These results were compared by visit type: new patient, or follow-up/postoperative.

**Figure 2:**
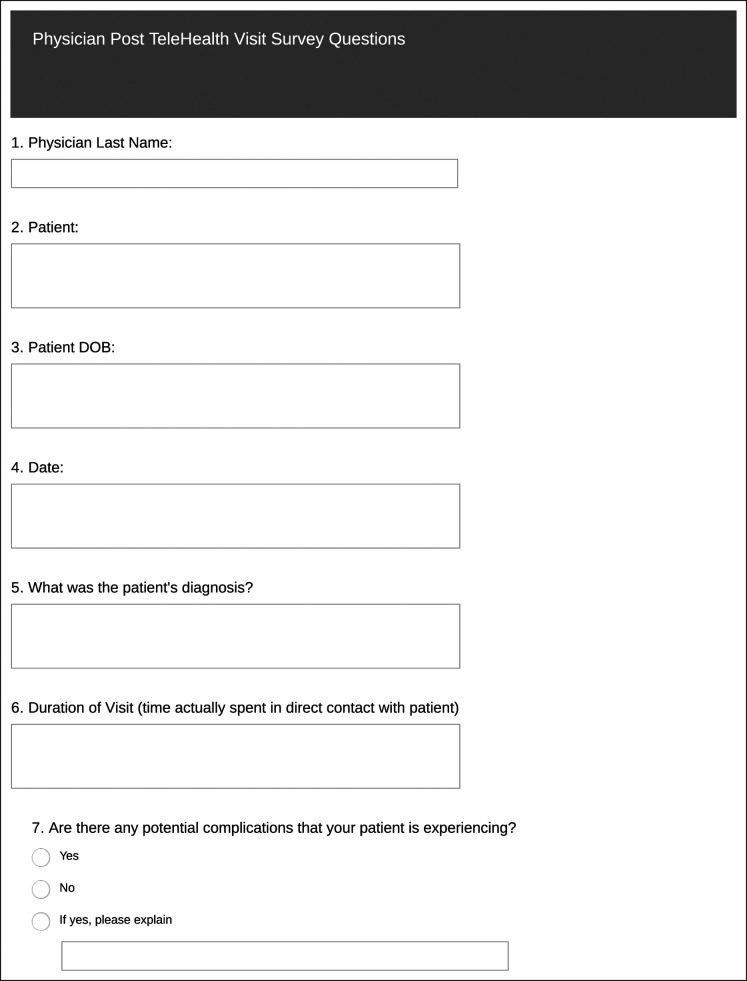

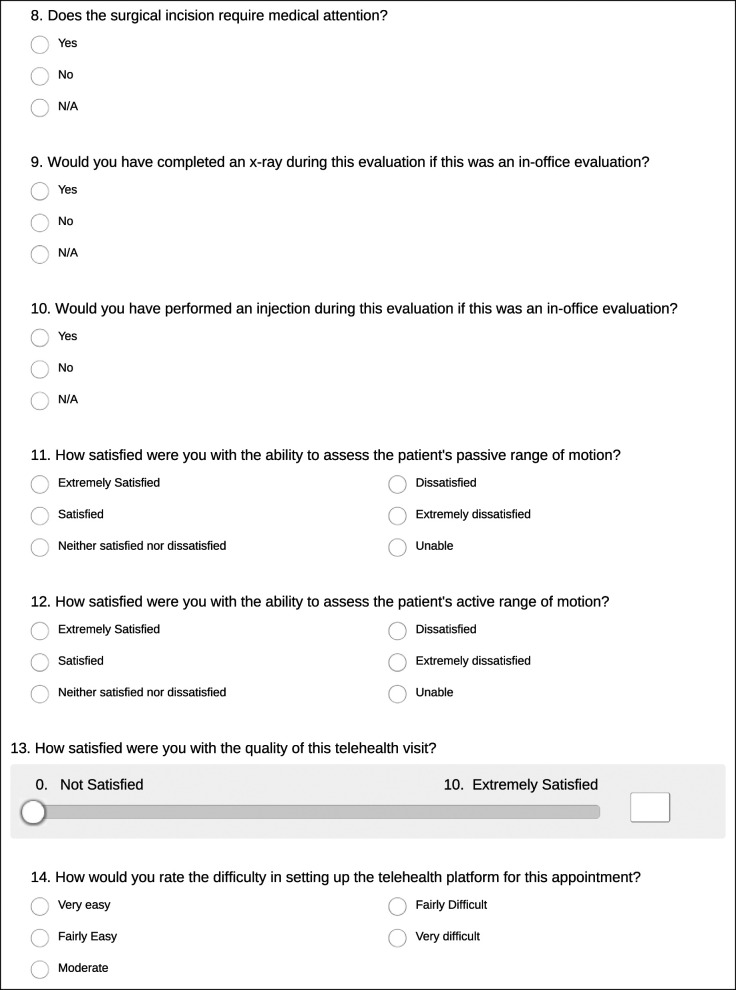

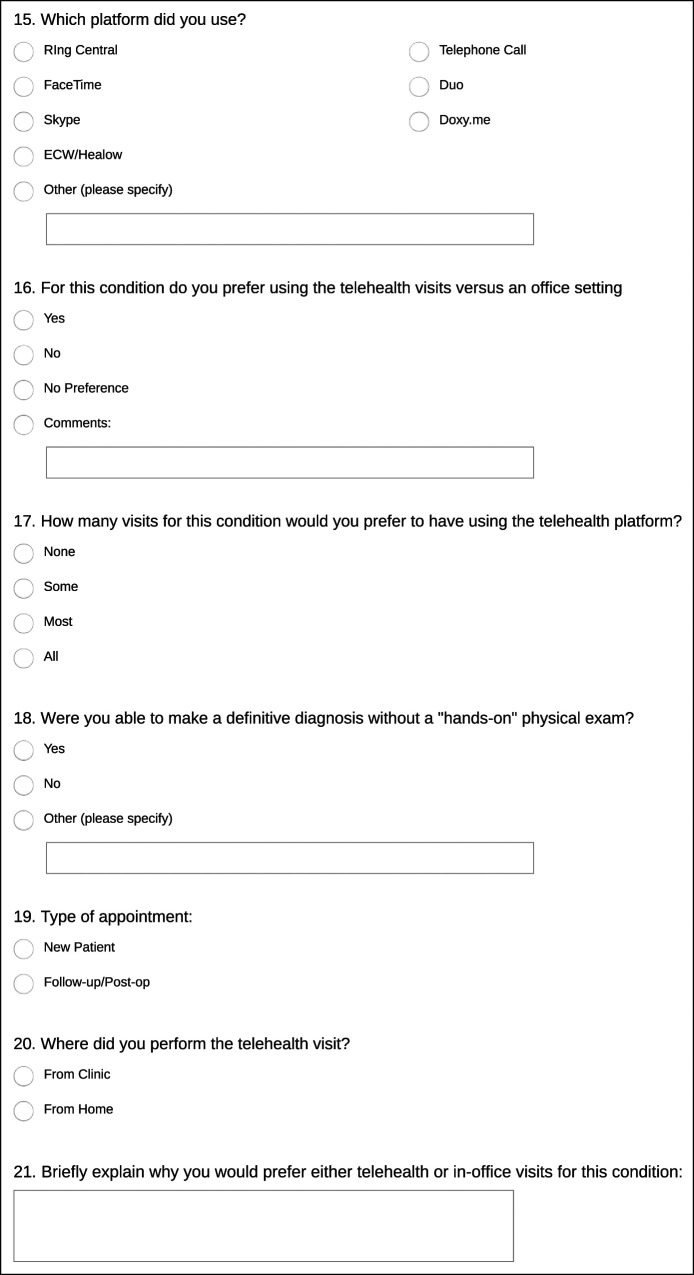
Chart showing physician survey.

The second part of the study consisted of a physician survey which was completed by the physician at the conclusion of the encounter. This physician survey (Figure 2) included ease of establishing an encounter, type of encounter, software/application used, perceived diagnostic accuracy of the encounter, and whether telehealth was preferred over in-person visits.

A sample size estimate revealed that roughly 200 surveys would need to be completed by both patients and physicians for this study to generate adequate power. Based on practice patterns, we predicted approximately 50% would include patient encounters with video connection, whereas the remaining visits would be completed by audio alone. For this analysis, the data were analyzed separately for patients and physicians. For patients, the data were first split between phone and video and then split by new patient and follow-up. For physicians, the data were split between new patient and follow-up. All continuous data are presented as mean (SD), and all categorical data are presented as cell count (percent of total count). Student *t*-tests were used to calculate *P* values for continuous data, and chi-square tests were used for categorical data. Significance was established at a *P* value of < 0.05. All statistical analyses were performed using R Studio (Version 3.6.3; Vienna, Austria).

## Results

A total of 180 patient surveys and 302 physician surveys were completed. Patient responses are listed in Table 1.

**Table 1:** Patient Data

Factor	*N* = 180
Age, yr, mean (SD)	56.0 (16.4)
Type of encounter, n (%)	
Telephone	77 (42.8)
Video	64 (35.6)
Did not answer	39 (21.7)
Was this visit for an/a? n (%)	
Existing issue/follow-up/post-op	111 (61.7)
New issue with this physician	56 (31.1)
Did not answer	13 (7.22)
How satisfied were you with your telehealth visit? n (%)	
Very satisfied	144 (80.0)
Somewhat satisfied	21 (11.7)
Neither satisfied nor dissatisfied	6 (3.33)
Somewhat dissatisfied	6 (3.33)
Dissatisfied	3 (1.67)
Do you feel the physician spent enough time with you? n (%)	
Yes	172 (95.6)
No	8 (4.44)
Were all of your questions or concerns addressed? n (%)	
Yes	171 (95.0)
No	8 (4.44)
Did not answer	1 (0.56)
Did your physician clearly communicate your care plan and follow-up instructions? n (%)	
Yes	173 (96.1)
No	7 (3.89)
How would you rate the technical difficulty participating in the telehealth visit? n (%)	
Very easy	125 (69.4)
Fairly easy	30 (16.7)
Moderate	14 (7.78)
Fairly difficult	7 (3.89)
Very difficult	2 (1.11)
Did not answer	2 (1.11)
Do you prefer to have visits using the home-based telehealth platform? n (%)	
Yes	88 (48.9)
No	89 (49.4)
Did not answer	3 (1.67)
How many of your visits would you prefer to have via the telehealth platform? n (%)	
All	5 (2.78)
Most	32 (17.8)
Some	119 (66.1)
None	23 (12.8)
Did not answer	1 (0.56)

### Patient Responses

Patients generally felt very satisfied (80%) with their encounter. Ninety-six percent felt the physician spent enough time with them, and 95% felt all of their concerns were addressed. Seventy percent felt the technical aspects of participating in the telehealth visit was very easy, with 17% responding it was fairly easy. Patients were evenly split on whether they preferred to have visits using the home-based platform. Sixty-six percent of patients answered the choice that they would like to have “some” of their doctor visits via the telehealth platform.

Sixty-three percent of new patients preferred telehealth compared with 45% of follow-up visit patients. When comparing these results based on the diagnosis, no statistical difference was observed (*P* = 0.07) related to new patient preference for telehealth. Satisfaction responses for these two groups were similar (*P* = 0.769). Patient responses for new versus follow-up or postoperative visits are listed in Table 2.

**Table 2:** Patient Responses, Existing Issue Versus New Issue

Factor	Existing Issue/Follow-up/Post-op	New Issue With This Physician	*P*
	*N* = 111	*N* = 56	
Age, yr, mean (SD)	57.9 (15.5)	53.7 (15.9)	0.125
How satisfied were you with your telehealth visit? n (%)			0.769
Very satisfied	88 (79.3)	46 (82.1)	
Somewhat satisfied	11 (9.91)	7 (12.5)	
Neither satisfied nor dissatisfied	5 (4.50)	1 (1.79)	
Somewhat dissatisfied	4 (3.60)	2 (3.57)	
Dissatisfied	3 (2.70)	0 (0.00)	
Do you prefer to have visits using the home-based telehealth platform? n (%)			0.071
Yes	50 (45.0)	35 (62.5)	
No	58 (52.3)	21 (37.5)	

The responses were compared between a telephone call and a video encounter. Patients found the technical difficulty to be lower with a phone call than the video platform. Sixty-one percent of those having a video encounter answered the technical aspects to be “very easy.” Fifty-eight percent of patients preferred telehealth if they had a video encounter versus 39% if they had a telephone call. This difference was statistically significant (*P* = 0.03). Patient responses for telephone versus video encounter are listed in Table 3.

**Table 3:** Patient Responses, Phone Versus Video

Factor	Phone	Video	*P*
	*N* = 77	*N* = 64	
Age, yr, mean (SD)	58.9 (14.9)	54.7 (15.9)	0.121
Do you prefer to have visits using the home-based telehealth platform? n (%)			**0.030**
Yes	30 (39.0)	37 (57.8)	
No	46 (59.7)	27 (42.2)	
How many of your visits would you prefer to have via the telehealth platform? n (%)			0.093
All	2 (2.60)	1 (1.56)	
Most	8 (10.4)	15 (23.4)	
Some	55 (71.4)	44 (68.8)	
None	11 (14.3)	4 (6.25)	

An analysis of the qualitative open-ended question “Briefly explain why you would prefer either telehealth or in-person office visits” saw several common themes. Those patients who preferred in-office visits noted it was usually because of perceived quality with an in-person examination. Some example responses included the following statements: I feel they get a better appreciation when they can be hands on especially with orthopedic issues. I would feel as if the Dr. could give a better “in person” exam.

Patients who chose telehealth over an in-person visit usually stated such as a matter of convenience. Some patients' responses follow: Easier to do and less time consuming. Doctor was on time and it went smoothly. I liked it because there's no waiting time. I prefer the telehealth visits because it was more relaxed, and I was in my home.

Others were thankful for this modality because of the pandemic but felt telehealth was a temporary measure not to replace the in-person interaction. Responses included: I am a people person, so I prefer face to face visits, but I think that the Telehealth visit is a good option for what is going on right now and for people who have a hard time getting to the office. Because of the COVID-19 virus telehealth is better for social distancing.

The last group saw telehealth as an additional modality to consider for selected conditions and at appropriate intervals in the treatment process. Responses in this group included: It depends on the nature of the problem. My concerns and questions were answered without a physical needed at this time. I feel that alternating between telehealth visits and in office visits would be perfect. I feel that the Dr. really listened to me and I was able to have all of my questions answered on telehealth. Although this is necessary at this time, nothing replaces the interaction between a patient and his/her doctor. Your confidence level increases when you are in the room with the doctor. Although my daughter's injury is minor, and I believe time will heal it as the doctor suggested, I would have been more confident in the results if he actually was able to examine her thumb. I feel a little more reassured going to an office for more involved visits. Routine follow-ups are ok for telehealth visits.

### Physician Responses

Physicians reported an average of just under 9 minutes in direct patient contact during all the telehealth visits. Surgical incision infrequently required medical attention, required in only 4 of 287 encounters (1%). For the ability to assess patient passive range of motion, the most common response was “extremely satisfied” (44%). Regarding active range of motion, the most common response was also “extremely satisfied” (55%). “Very easy” was selected most frequently (76%) regarding setting up each telehealth visit. Because our practice just recently began using this modality, several different platforms were used. A telephone call was used 42% of the time. The other 58% of the encounters were done over video with eClinicalWorks (22% of the overall) and then Doxy.me (18% of the overall). Combining all types of patient encounters, the physicians responded that half of the time they would prefer telehealth over an in-person visit. Seventy-three percent of the time they felt “some” would be the best response to how many visits during treatment should be done with the telehealth modality. Eighty-four percent of the time they felt they could make a definitive diagnosis without a hands-on physical examination.

New patients and follow-up/postoperative patient visits are reported in Table 4. New patient visits were just over 11 minutes, and follow-up visits were just over 8 minutes. Providers felt that a radiograph was needed in 53% of new patients versus 18% for follow-up visits (*P* < 0.001). Providers would have performed an injection in 51% of new patients versus 24% for follow-up visits (*P* < 0.001). For new patient visits, only 23% (19 of 81) would not have needed a radiograph or injection optimally at the time of visit. When asked their preference for performing visits for the patient's condition via telehealth, 40% responded “yes” for new visits and 53% responded “yes” for follow-ups (*P* ≤ 0.004).

**Table 4:** Physician Data

Factor	Follow-up/Post-op	New Patient	*P*
	*N* = 206	*N* = 81	
Age, yr, mean (SD)	53.2 (16.9)	50.4 (17.4)	0.225
Time spent in direct contact with patient, min, mean (SD)	8.09 (3.20)	11.2 (3.90)	<0.001
Potential complications, n (%)			0.016
Yes	22 (10.7)	1 (1.23)	
No	184 (89.3)	80 (98.8)	
Medical attention needed for surgical wound, n (%)			<0.001
Yes	4 (1.94)	0 (0.00)	
No	117 (56.8)	18 (22.2)	
N/A	85 (41.3)	63 (77.8)	
Was an radiograph needed? n (%)			<0.001
Yes	37 (18.0)	43 (53.1)	
No	166 (80.6)	37 (45.7)	
N/A	2 (0.97)	1 (1.23)	
Injection needed? n (%)			<0.001
Yes	50 (24.3)	41 (50.6)	
No	155 (75.2)	39 (48.1)	
N/A	0 (0.00)	1 (1.23)	
Assess active range of motion, n (%)			0.256
Extremely satisfied	119 (57.8)	38 (46.9)	
Satisfied	55 (26.7)	27 (33.3)	
Neither satisfied nor dissatisfied	21 (10.2)	8 (9.88)	
Dissatisfied	2 (0.97)	2 (2.47)	
Extremely dissatisfied	0 (0.00)	1 (1.23)	
Unable	9 (4.37)	5 (6.17)	
Satisfaction with quality of visit, mean (SD)	87.4 (14.5)	85.2 (13.6)	0.224
Difficulty in setting up the telehealth platform, n (%)			0.102
Fairly difficult	7 (3.40)	8 (9.88)	
Fairly easy	26 (12.6)	8 (9.88)	
Moderate	11 (5.34)	5 (6.17)	
Very difficult	1 (0.49)	2 (2.47)	
Very easy	161 (78.2)	58 (71.6)	
Platform used, n (%)			0.006
Doxy.me	37 (18.0)	16 (19.8)	
eClinicalWorks/healow	45 (21.8)	28 (34.6)	
FaceTime	8 (3.88)	4 (4.94)	
RingCentral	13 (6.31)	8 (9.88)	
Telephone call	100 (48.5)	21 (25.9)	
Other	3 (1.46)	4 (4.94)	
For this condition, do you prefer using the telehealth visits versus an office setting? n (%)			0.004
Yes	110 (53.4)	32 (39.5)	
No preference	7 (3.40)	9 (11.1)	
No	84 (40.8)	35 (43.2)	
Other	3 (1.46)	5 (6.17)	
How many visits for this condition would you prefer to have using the telehealth platform? n (%)			0.300
All	0 (0.00)	1 (1.23)	
Most	28 (13.6)	14 (17.3)	
Some	155 (75.2)	55 (67.9)	
None	22 (10.7)	10 (12.3)	
Definitive diagnosis without a hands-on physical examination, n (%)			0.063
Yes	180 (87.4)	62 (76.5)	
Other (please specify)	3 (1.46)	2 (2.47)	
No	23 (11.2)	17 (21.0)	

N/A, not applicable.

Physicians were able to make a definitive diagnosis with a telehealth physical examination in 87% of follow-up/postoperative and 77% for new patient encounters (*P* = 0.06). The overall physician satisfaction was 85% and 87%, respectively, for new and follow-up/postoperative patient encounters.

Physician comments to the question “Briefly explain why you would prefer either telehealth or in-office visits for this condition” included statements regarding diagnostic accuracy, convenience, and patient safety. Patient safety in the current pandemic was the reason most often cited for the telehealth visit. Physicians felt certain conditions were hard to evaluate over the phone, and if an radiograph or injection was needed, then the patient should be seen in person.

## Discussion

Telehealth is the use of telecommunication and information technology to provide healthcare remotely without direct physical patient contact. The use of this technology has the potential to provide safe patient access to healthcare while minimizing travel, reducing time taken off from work or school, and eliminating direct patient exposure during a health crisis. There has been an exponential increase in the utilization of telehealth in orthopaedic surgery during the COVID-19 pandemic becuase this modality eliminates the risks associated with face-to-face interaction while still providing satisfactory and efficient patient care.^3^ Although a fair amount of existing literature exploring the use of telehealth exists, the recent COVID-19 pandemic has exponentially increased the opportunity to explore this modality to provide safe and quality orthopaedic care.

Numerous authors have looked at the use of telehealth for postoperative follow-up in orthopaedics. Daruwalla et al^7^ found that the utilization of a remote presence robotic system in an orthopaedic postoperative care setting resulted in very positive reactions from both patients and nursing staff as obtained with questionnaires. Marsh et al evaluated the role of telemedicine in patients after total joint arthroplasty by randomizing them to complete either web-based follow-up or in-person follow-up in clinic 12 months postoperatively. Web-based follow-up was proven to be a feasible, clinically effective alternative to in-person visits, with a lower associated cost.^8^ Of note, although the patients reported moderate to high levels of satisfaction with telemedicine, patients completing standard in-person visits did report slightly higher satisfaction.^9^ Conversely, a study by Sharareh and Schwarzkopf^10^ reported that telemedicine visits after total joint arthroplasty were associated with increased patient satisfaction compared with those who underwent standard in-person follow-up. In addition, in a report by Abel et al^11^ after adolescent patients after knee arthroscopy, two-thirds of the subjects preferred telemedicine visits after having both a video and in-clinic postoperative visit.

Kane et al found that patients undergoing arthroscopic rotator cuff surgery were able to receive safe and effective early postoperative follow-up care using telemedicine. In this prospective randomized controlled trial, patients were assigned to complete either in-office or telemedicine follow-up after undergoing arthroscopic rotator cuff repair. Patients in both groups experienced similar pain scores and satisfaction scores, and in the telemedicine group, preference for telehealth increased from the baseline for both surgeons and patients after first-hand experiences with this modality. This study also documented an additional benefit of telehealth that visits required less time off from work for both patients and caregivers.^12^

Regarding the role of telemedicine in orthopaedic consultation, a study by Buvik et al in 2018 observed that no difference was observed in patient-reported satisfaction and health outcomes between subjects randomized to an in-person consultation versus a video visit. It was also noted in this study that 63% of patients randomized to the in-person consultation and 86% of patients in the video-visit group both preferred to use telemedicine for subsequent encounters.^13^ A study by Abboud et al looked at the reliability of telemedicine consultation for patients with upper extremity problems. Patients underwent initial evaluation by an independent evaluator, which consisted of in-person history, physical examination, and imaging. This information was electronically presented 6 months later to two hand surgeons, who formulated diagnosis and treatment plans. When comparing the findings, the telemedicine consultation had excellent agreement within and between observers.^14^

When focusing on patients with hand and upper extremity injuries specifically, previous studies have looked at the use of telemedicine for emergency consultations in this population. In 2014, the Hand Trauma Telemedicine Program was implemented in Arkansas to address limited access to subspecialists. It was found that using telemedicine was successful in yielding a notable decrease in the amount of unnecessary transfers for patients with hand problems, reducing healthcare costs and improving the efficiency of specialized care.^15^

Regarding the cost-effectiveness of telemedicine in orthopaedics, Harno et al^16^ reported that teleconsultation costs 45% less than in-person outpatient orthopaedic visits when considering equipment costs, maintenance, and staffing. Additional reports have shown cost savings about travel and societal expenses with the implementation of remote orthopaedic consultations.^17,18^ A systematic review by Torre-Diaz et al looked at the cost utility and cost effectiveness of telemedicine across all fields and found that a relative scarcity of the literature is seen on this topic. Some cost effectiveness studies reported that telemedicine was able to reduce costs, but not all supported this notion.^19^ It has also been noted that although telehealth visits cost less than in-person visits, the convenience and availability of direct-to-consumer telehealth could potentially drive overall increased utilization and healthcare spending.^20^

The implementation of telehealth in orthopedics can potentially benefit both the patient and the provider. For physicians, this technology may allow for increased patient volume and an expansion in the catchment area for patient referrals. It may also decrease overhead costs, decrease the need for auxiliary staff, allow for working off hours without the requirements of additional staffing, and free up office space if multiple providers are sharing a workspace. In addition, it can limit physician travel between offices. If this technology is to be integrated into a practice, telemedicine encounters may be scheduled by a time slot, similar to standard in-person visits. However, it is unknown whether patients will feel comfortable eventually having a copay or being charged regular rates for a telehealth visit. If telemedicine encounters can yield outcomes that are similar to face-to-face visits, with high levels of patient satisfaction, it may be reasonable for patients to be charged the same rate as a standard visit. Patients will have experienced a similar 10-minute encounter with the physician while being saved the trouble of travel, check in, and wait time. On the other hand, some patients may feel that the patient-physician relationship is being compromised with this technology. Although a video connection can feel personal to an extent, it cannot take the place of a face-to-face interaction, especially when hands-on examination is involved. This prospective study has the advantage of capturing both patient and physician perception of a telehealth encounter to provide a more comprehensive picture of the utility of this modality for future patient care.

In this study, a mixed response was observed from patients as to whether they would prefer to have visits using the home-based telehealth platform. There was a perception that “hands-on” care was associated with higher quality care. Those who preferred telehealth usually appreciated the convenience provided by using this technology. Clearly, patients were grateful that patient-physician relationships could be maintained during the pandemic.

There are several limitations to this study. First, over 40% of the patient surveyed had a consultation that was conducted by a telephone call without video connection. It is possible that the addition of a video connection in a greater proportion of these visits could have led to increased patient satisfaction because this would have allowed for the physician to visually examine the upper extremity. It may have also made the interaction more similar to a standard face-to-face experience for patients. However, even video conferencing would not be able to address the lack of hands-on examination and potential intervention such as radiograph or injection, with these encounters. Second, inconsistency was observed in the frequency with which physicians and patients completed surveys because not every patient encounter throughout the duration of this study was followed by completion of a survey.

Although telehealth cannot take the place of a hands-on patient interaction, there is undoubtedly a time and place for its implementation in orthopedics. Although this modality proved to be extraordinarily useful during the COVID-19 pandemic, it can also have utility in a time without mandated restrictions on in-person interaction. The extent of the incorporation of this modality into orthopaedic practice remains to be determined, along with the most efficient and practical method of implementation. Tanaka et al^21^ recently produced a protocol for virtual visits and examination in orthopedics. This can serve as a guideline and starting point for physicians who are beginning to implement telemedicine and can be tailored to the practice of each individual provider. Although telemedicine can be used to evaluate and triage new problems, it may be best suited for follow-up and postoperative visits because of the more probable need to perform radiographs or injections during a new patient visit. It can potentially be feasible to offer patients the choice, if deemed appropriate by the provider, whether they would prefer in-person or telemedicine follow-up visits. For patients who travel a notable distance to receive care, have physical difficulties with travel, or who have difficulty taking time away from work, school, or other obligations, this may be a convenient and preferable option. Continued exploration and understanding of patient and physician perception of telehealth visits can guide the use of this tool for optimal benefits in the field of orthopedics.
